# Influence of proton pump inhibitors and histamine H_2_ receptor antagonists on serum phosphorus level control by calcium carbonate in patients undergoing hemodialysis: a retrospective medical chart review

**DOI:** 10.1186/s40780-016-0068-1

**Published:** 2016-11-22

**Authors:** Masaomi Tatsuzawa, Ryuichi Ogawa, Atsushi Ohkubo, Kazuyo Shimojima, Kunimi Maeda, Hirotoshi Echizen, Akihisa Miyazaki

**Affiliations:** 1 Department of Pharmacy, Juntendo University Nerima Hospital, 3-1-10 Takanodai, Nerima-ku, Tokyo, 177-8521 Japan; 2 Department of Pharmacotherapy, Meiji Pharmaceutical University, 2-522-1 Noshio, Kiyose-shi, Tokyo, 204-8588 Japan; 3 Department of Nephrology, Juntendo University Nerima Hospital, 3-1-10 Takanodai, Nerima-ku, Tokyo, 177-8521 Japan; 4 Departments of Gastroenterology and Pharmacy, Juntendo University Nerima Hospital, 3-1-10 Takanodai, Nerima-ku, Tokyo, 177-8521 Japan

**Keywords:** Drug interactions, Proton pump inhibitors, Histamine H_2_ receptor antagonists, Calcium carbonate, hemodialysis, Retrospective studies

## Abstract

**Background:**

Hyperphosphatemia is one of the common complications in patients undergoing hemodialysis. Although calcium carbonate (CaC) is often used to control serum inorganic phosphorus level in dialysis patients, co-administration of gastric acid reducers (ARs) may interfere with the phosphate binding effect of CaC. We performed a retrospective medical chart review to study whether ARs attenuate the hypophosphatemic effect of CaC in patients undergoing hemodialysis.

**Methods:**

One hundred and eight chronic hemodialysis patients receiving either CaC alone or CaC concomitant with one of the ARs (proton pump inhibitors and histamine H_2_-receptor antagonists) were retrieved from the medical charts in Juntendo University Nerima Hospital. The patients were subdivided according to the interval between hemodialysis sessions (interdialysis interval of 48 or 72 h). A multivariate analysis was performed to identify clinical covariates associated with the variability of serum inorganic phosphorus levels. The study protocol was approved by the Institutional Review Board before the study was begun.

**Results:**

Among patients on hemodialysis with a 72-h interdialysis interval, the magnitude of increase in serum inorganic phosphorus concentration in patients receiving CaC and AR was significantly greater than in those receiving CaC alone. While a similar trend was observed among patients with a 48-h interdialysis interval, the difference did not reach a significant level. A multivariate regression analysis revealed that concomitant administration of ARs with CaC and a longer interdialysis interval (72 h) were significantly and independently associated with the magnitude of increase in serum phosphorus concentration between dialysis sessions. No significant differences in albumin-corrected serum calcium concentrations and incidence of pathological fractures were observed between patients receiving CaC alone and those receiving CaC with ARs.

**Conclusions:**

Concomitant use of ARs with CaC may attenuate the hypophosphatemic effect of CaC in patients undergoing chronic hemodialysis. When hemodialysis patients require prescription of ARs for the prevention of upper gastrointestinal mucosal diseases (such as peptic ulcer), it may be prudent to choose a phosphate binder other than CaC.

## Background

Hyperphosphatemia is one of the common complications in patients with end-stage renal disease (ESRD) undergoing hemodialysis. Impaired renal phosphorus excretion causes hyperphosphatemia and may lead secondarily to hypocalcemia according to the physicochemical inverse equilibrium between phosphorus and calcium ions. Hypocalcemia triggers secondary hyperparathyroidism that may ultimately result in renal osteodystrophy and pathological fracture as well as ectopic calcification [1]. Collectively, hyperphosphatemia alone or in combination with hypercalcemia has been associated with increased cardiovascular and musculoskeletal morbidity and mortality in hemodialysis patients [2, 3]. In this context, management of serum inorganic phosphorus level is clinically important. Restriction of dietary phosphate intake, oral administration of phosphate binder, and more intensive hemodialysis are commonly recommended for the prevention and treatment of hyperphosphatemia in patients undergoing chronic hemodialysis. As for the choice of phosphate binder, calcium carbonate (CaC) may be preferred to lanthanum carbonate or sevelamer hydrochloride when safety profile or lower cost is of primary concern.

Because ESRD patients commonly take low-dose aspirin for the prevention of atherosclerotic cardiovascular diseases [4–6], they often develop acid-related upper gastrointestinal diseases (including peptic ulcer and reflux esophagitis) [7] and frequently take one of the proton pump inhibitors (PPIs) or histamine H_2_ receptor antagonists (H_2_RAs). There is a concern that administration of these gastric acid reducers (ARs) may interfere with the phosphate binding effect of CaC [8–11]. The dissolution rate of tablet formulation of precipitated CaC is strongly pH dependent. *In vitro* dissolution tests demonstrated a mean dissolution rate of 99.7% within 10 min at pH 1.2, but only 10.9% by 360 min at pH 6.8 [12]. As a result, concomitant administration of ARs with CaC may attenuate the dissolution of CaC formulation, consequently releasing less free calcium ions to bind phosphate ions derived from food [12–14]. Previous clinical studies, however, have reported controversial results regarding the interaction between ARs and CaC [15, 16].

We hypothesize that different study designs adopted in previous studies may account for the contradictory results obtained for the interaction between ARs and CaC. There are large variations in pre- and post-hemodialysis serum inorganic phosphorus concentrations and in the interval between hemodialysis sessions among patients. As a result, the interaction would be most effectively studied by comparing serum inorganic phosphorus concentrations using paired data from the same patients and by considering the length of hemodialysis intervals (usually either 48 or 72 h). Theoretically, the effect of drug interaction between ARs and CaC is greater as the interval between hemodialysis sessions (interdialysis interval) increases. In this study, we performed a retrospective medical chart review to analyze the interaction between ARs and CaC based on the change in serum inorganic phosphorus concentration from after dialysis to just before dialysis session in the same patients, adjusting for the interdialysis interval.

## Methods

### Study design and data retrieval

The present study was performed by retrospectively reviewing patients’ data extracted from electronic medical records archived at Juntendo University Nerima Hospital, Japan. First, ESRD patients undergoing maintenance hemodialysis from January 2006 to December 2014 were retrieved from the electronic medical records. Patients who received CaC for the treatment of hyperphosphatemia were extracted, and those taking either sevelamer or lanthanum with CaC were excluded. From the medical records, those that had blood chemistry data obtained immediately after and before hemodialysis within 1 month under stable and comparable dialysis conditions in the same patients were considered eligible data sets. Patients with variable hemodialysis conditions during the study period were excluded. The paired data of each patient were searched chronologically over the study period, and the earliest pair of post- and pre-hemodialysis (post-HD and pre-HD) data obtained within 1 month was collected. The patients were stratified according to the interdialysis interval (48 or 72 h). It should be noted that the pair of post-HD and pre-HD data set were not necessarily collected at an interval of 48 or 72 h, because serum data obtained after a hemodialysis session was paired with those obtained before a session sometime within 1 month. One data set pair was obtained from one patient. CaC was prescribed as a tablet formulation (500 mg) of precipitated CaC (Sanwa Kagaku Kenkyusho Co., Ltd.). While pharmacists instructed patients to ingest the CaC tablet during meal, they could not confirm if the patients adhered to the instruction. Dialysate contained sodium (140 mEq/L), potassium (2.0 mEq/L), calcium (3.0 mEq/L), magnesium (1.0 mEq/L), chloride (111 mEq/L), bicarbonate (35 mEq/L) and glucose (1.5 g/L). Cellulose triacetate dialyzers and polysulfone dialyzers were used in approximately 70% and 30% of the patients, respectively. The protocol of the present study was written according to the ethics guidelines for clinical studies and was reviewed and approved by the Institutional Review Board at Juntendo University Nerima Hospital (reference number: 27-04).

### Assessment of interaction between AR and CaC

Patients were divided into two groups: those taking ARs with CaC (the AR + CaC group) and those taking CaC only (the CaC group). Glomerular filtration rate was calculated by the Modification of Diet in Renal Disease (MDRD) equation for Japanese [17] using serum creatinine levels obtained before hemodialysis session. We assessed whether the hypophosphatemic effect of CaC is affected by co-administration with ARs by comparing the difference between post-HD and pre-HD serum inorganic phosphate levels in the same patients. The statistical analyses were performed for the data combining those obtained from patients irrespective of dialysis intervals (i.e., 48 h or 72 h) together (the main analysis) and for those stratified into 48-h and 72-h dialysis intervals separately (the subgroup analysis). For analyzing whether there is a difference in the magnitude of anti-hypophosphatemic effect between ARs, we compared the changes in serum phosphate concentrations during dialysis intervals obtained from patients given different ARs. We also examined whether there is an imbalance in the dialysis intervals for each AR data. Furthermore, we also examined whether coadministration of ARs with CaC influences albumin-corrected serum calcium level or the incidence of fracture events in the AR + CaC and CaC groups. Fracture was diagnosed by plain X-ray findings, together with computed tomography findings if necessarily.

### Statistical analysis

Student’s *t*-test was used to compare the mean values of continuous variables (such as age) between the AR + CaC and CaC groups. Chi-square test or Fisher’s exact test was used for comparisons of categorical variables (such as sex). One-way ANOVA was employed for multiple comparisons of changes in serum phosphate levels during the interdialysis period irrespective of the dialysis intervals (i.e., 48 and 72 h) in patients who received different ARs with CaC. For the subgroup analysis assessing the effects for 48-h and 72-h dialysis intervals separately, we employed a post-hoc Bonferroni’s correction (k = 2) for suppressing overall the type I error <0.05. Comparisons of the magnitude of the drug interaction between different ARs and CaC were made using ANOVA after heterogeneity in the distribution of dialysis intervals across different ARs was examined by the Fischer’s exact test. A multiple regression analysis was performed to examine whether co-administration of ARs and other clinical covariates (sex, age, dry weight, daily dose of CaC, interdialysis interval, and residual renal function assessed by glomerular filtration rate) are associated with the variability of serum inorganic phosphorus levels. We used JMP Pro 11 (SAS Institute Inc., North Carolina, USA) for all statistical analyses, and chose 0.05 as the critical value of the type I error throughout the study. Data are expressed as mean ± standard deviation, median [upper and lower quartile values], or number of patients (percentage).

## Results

### Patients

**Fig. 1:**
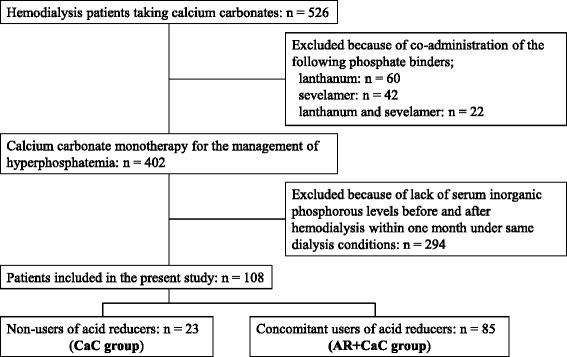
Flow chart of patient selection. CaC, calcium carbonate; AR, acid reducer

Among the 526 hemodialysis patients taking CaC as a phosphate binder at Juntendo University Nerima Hospital during the study period, 108 patients were eligible to enter the present study according to the inclusion and exclusion criteria (Fig. 1). Among them, 23 patients (aged 66 ± 12 years, 14 males) were assigned to the CaC group and 85 (aged 70 ± 12 years, 55 males) to the AR + CaC group. Thirty-six patients (9 and 27 in the CaC and the AR + CaC group, respectively) and 72 patients (14 and 58 in the CaC and the AR + CaC group, respectively) had interdialysis intervals of 48 and 72 h, respectively. Table 1 shows the patients’ demographic data, laboratory tests, underlying diseases and concomitant medications. While the mean daily dose of CaC in the AR + CaC group (2.5 ± 1.3 g/day) tended to be higher than that in the CaC group (1.9 ± 1.1 g/day), the difference did not reach statistical significance (*p* = 0.062). When the CaC doses were compared between the CaC and AR + CaC groups for interdialysis intervals of 48 and 72 h separately, essentially similar results were obtained (data are not shown). No significant difference in the percentage of patients receiving vitamin D_3_ analogues was observed between the CaC and AR + CaC groups (*p* = 0.466). No significant differences were observed between two groups in biochemical data (including pre-HD creatinine and blood urea nitrogen) possibly related to hemodialysis conditions.

**Table 1:** Demographic and laboratory data of patients studied

Characteristics	CaC group	AR + CaC group	*P* value
Number of patients, n	23	85	-
Male, n (%)	14 (61)	55 (65)	0.734
Age, years	66 ± 12	70 ± 12	0.172
Total body weight, kg	56.3 ± 9.3	53.8 ± 12.1	0.362
Dry weight, kg	54.9 ± 9.1	52.3 ± 12.0	0.347
Dialysis time, min	199 ± 27	212 ± 28	0.060
Dose of calcium carbonate, g/day	1.9 ± 1.1	2.5 ± 1.3	0.062
Pre-HD blood urea nitrogen, mg/dL	53.0 ± 13.2	54.2 ± 17.9 [*n* = 83]	0.771
Pre-HD serum creatinine, mg/dL	7.53 ± 2.24	8.26 ± 2.77	0.246
Pre-HD estimated GFR, mL/min/1.73 m^2^	6.2 ± 2.0	5.9 ± 2.6	0.561
Serum albumin, g/dL	3.0 ± 0.6 [*n* = 20]	3.3 ± 0.6 [*n* = 80]	0.152
Alkaline phosphatase, IU/L	437 ± 336 [*n* = 10]	304 ± 188 [*n* = 20]	0.266
Intact parathyroid hormone, pg/mL	25 [*n* = 1]	93.8 ± 84.7 [*n* = 14]	-
Post-HD serum inorganic phosphorus, mg/dL	2.3 ± 0.7	2.1 ± 0.7	0.329
Post-HD serum calcium, mg/dL	8.8 ± 0.9 [*n* = 22]	9.0 ± 0.6 [*n* = 84]	0.238
Post-HD corrected serum calcium, mg/dL	9.6 ± 1.0 [*n* = 19]	9.6 ± 0.6 [*n* = 84]	0.984
*Co-morbidities*			
Hypertension, n (%)	17 (74)	62 (73)	0.926
Dyslipidemia, n (%)	2 (9)	16 (19)	0.279
Type 2 diabetes, n (%)	14 (61)	40 (47)	0.240
Heart disease, n (%)	8 (35)	40 (47)	0.293
Peptic ulcer disease, n (%)	0 (0)	6 (7)	-
Mineral and bone disorder, n (%)	4 (17)	11 (13)	0.626
Parathyroid disorder, n (%)	5 (22)	8 (9)	0.105
*Gastric acid reducers*			
Proton pump inhibitors, n (%)	-	73 (86)	-
Histamine H_2_ receptor antagonists, n (%)	-	12 (14)	-
*Osteoactive drugs*			
Vitamin D_3_ analogues, n (%)	5 (22)	24 (28)	0.533
Bisphosphonates, n (%)	0 (0)	0 (0)	-
Calcium sensitizers, n (%)	0 (0)	0 (0)	-

Data are expressed as mean ± standard deviation, or number (percentage)

*AR* acid reducer, *CaC* calcium carbonate, *GFR* glomerular filtration rate, *HD* hemodialysis

### Serum inorganic phosphorus levels

**Fig. 2:**
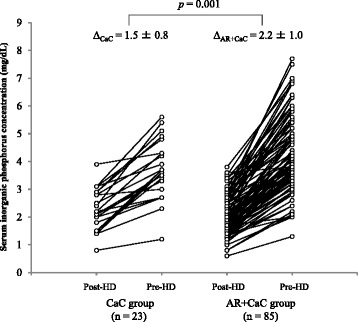
Changes in serum inorganic phosphorus levels of paired samples collected after dialysis and just before dialysis session. CaC, calcium carbonate; AR, acid reducer; HD, hemodialysis

**Fig. 3:**
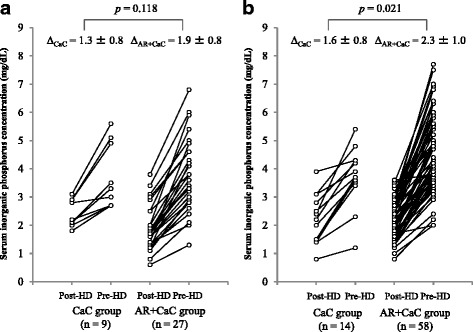
Changes in serum inorganic phosphorus levels of paired samples collected after dialysis and just before dialysis session in patients undergoing hemodialysis with 48-h interdialysis interval (**a**) and 72-h interdialysis interval (**b**). Bonferroni’s corrections were applied for the *p*-values. CaC, calcium carbonate; AR, acid reducer; HD, hemodialysis

Figure 2 shows serum inorganic phosphorus levels obtained from patients in the CaC and AR + CaC groups before and after hemodialysis session (post-HD and pre-HD, respectively). The data obtained from the same patients are connected by lines. The mean change in serum inorganic phosphate obtained from the AR + CaC group (2.2 ± 1.0 mg/dL) was significantly (*p* = 0.001) greater than that obtained from the CaC group (1.5 ± 0.8 mg/dL). Next, the patients were stratified into 48-h and 72-h interdialysis intervals, and the data of the AR + CaC and CaC groups were compared for each interval. For the 72-h interdialysis interval, the mean change in serum inorganic phosphorus concentration was significantly greater in the AR + CaC group than in the CaC group (2.3 ± 1.0 vs. 1.6 ± 0.8 mg/dL, *p* = 0.021, Fig. 3b). For the 48-h interdialysis interval, while the mean change in serum inorganic phosphorus concentration tended to the higher in the AR + CaC group than in the CaC group, the difference did not reach a statistical significance (1.9 ± 0.8 vs. 1.3 ± 0.8 mg/dL, *p* = 0.118, Fig. 3a). Multiple regression analysis identified concomitant use of ARs with CaC and interdialysis interval of 72 h as significant (*p* = 0.002 and 0.043, respectively) independent variables associated positively with the variability in changes of serum inorganic phosphorus level during the interdialysis interval, while the other covariates (sex, age, dry weight, daily dose of CaC, and residual glomerular filtration rate; Table 2) were not significantly associated. Daily dose of CaC was not significantly associated with the variability of change in serum inorganic phosphorus during the interdialysis interval.

**Table 2:** Results of multiple regression analysis investigating the association of various clinical covariates with interpatient variability in the magnitude of increase in serum inorganic phosphorus concentration during the interdialysis interval in Japanese hemodialysis patients (*n* = 108)

Covariate	PRC	SE	SPRC	VIF	P
Intercept	1.56	0.95	0.0	-	0.103
Concomitant use of AR	0.374	0.119	0.313	1.21	0.002
Female	0.116	0.112	0.114	1.49	0.306
Age (years)	−0.00802	0.00815	−0.102	1.31	0.327
Dry weight (kg)	0.0124	0.0108	0.145	1.93	0.252
Daily dose of CaC (g/day)	0.0357	0.0746	−0.0473	1.19	0.633
Interdialysis interval of 72 h	0.206	0.100	0.198	1.14	0.043
Residual GFR (mL/min/1.73 m^2^)	0.0060	0.0410	0.0167	1.60	0.885

*AR* acid reducers, *CaC* calcium carbonates, *GFR* glomerular filtration rate, *PRC* partial regression coefficient, *SE* standard error of PRC, *SPRC* standardized partial regression coefficient, *VIF* variance inflation factors

### Effect of different ARs on serum inorganic phosphorus levels

**Fig. 4:**
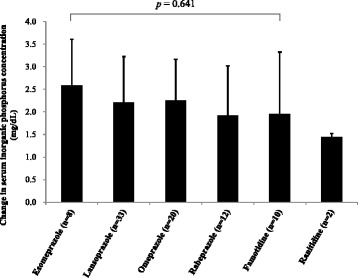
Effects of different ARs on change in serum inorganic phosphorus concentration. Error bar indicates standard deviation. AR, acid reducer

In the AR + CaC group, 73 and 12 patients were prescribed one of the PPIs (8 esomeprazole [20 mg/day], 33 lansoprazole [18 ± 6 mg/day], 20 omeprazole [20 ± 2 mg/day], and 12 rabeprazole [11 ± 3 mg/day]) and one of the H_2_RAs (10 famotidine [12 ± 4 mg/day], 2 ranitidine [150 mg/day]), respectively. No significant heterogeneity was observed regarding hemodialysis intervals (48 h vs 72 h) across different ARs (*p* = 0.101). Multiple comparisons showed no significant difference in the increase in serum inorganic phosphorus level during the interdialysis interval among the ARs examined, excluding ranitidine. Because only 2 patients were given ranitidine, these patients were excluded from comparison (Fig. 4).

### Corrected serum calcium levels

**Fig. 5:**
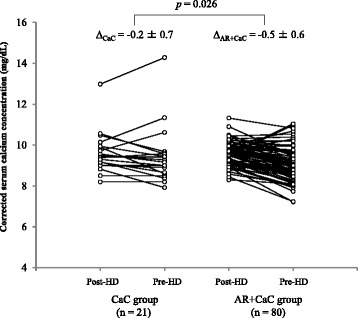
Changes in albumin-corrected serum calcium levels of paired samples collected after dialysis and just before dialysis session. CaC, calcium carbonate; AR, acid reducer; HD, hemodialysis

Figure 5 shows changes in corrected serum calcium level in post-HD and pre-HD measurements in the CaC and AR + CaC groups. The AR + CaC group showed significantly greater decrease in corrected serum calcium level compared to the CaC group (*p* = 0.026).

### Fracture events

Fracture events were searched by reviewing medical records throughout the study period for the patients analyzed. There was no significant difference in incidence of fracture events between the CaC and AR + CaC groups: 5 events out of 23 (22%) and 19 events out of 85 (22%) patients in the CaC and AR + CaC groups, respectively.

## Discussion

In the present study we revealed that concomitant administration of ARs with CaC significantly attenuated the hypophosphatemic effects of CaC in Japanese patients, particularly in those undergoing hemodialysis with a 72-h interdialysis interval. Our finding is consistent with that of Matsunaga et al. [10] from an observational study in 115 hemodialysis patients. They reported that the average pre-hemodialysis serum inorganic phosphorus level measured over 2 months after the initiation of concomitant treatment with CaC and famotidine (10 mg/day) or lansoprazole (30 mg/day) was significantly higher than the respective baseline value obtained with CaC alone [famotidine: 5.79 ± 1.17 vs. 5.55 ± 1.21 mg/dL (*p* < 0.05); lansoprazole: 5.80 ± 0.98 vs. 5.36 ± 1.39 mg/dL (*p* < 0.05)]. In contrast, Hardy et al. [15] conducted a prospective crossover trial in 16 hemodialysis patients treated with CaC alone for the initial 2 months and then switched to concomitant CaC and omeprazole (20 mg/day) for 2 months. They found no significant difference in mean serum inorganic phosphorus level between CaC alone (1.80 ± 0.38 mM) and CaC with omeprazole (1.89 ± 0.42 mM). At present, we cannot explain the discrepancies in findings described above. However, we assume that many clinical covariates are associated with inter-patient variability of serum inorganic phosphorus concentrations and their relative contribution to overall variability would be different among patients. In this context, our study design that compares the magnitude of increase in serum inorganic phosphorus levels during the interdialysis period in the same patients have allowed more sensitive detection of the interaction between ARs and CaC.

In the present study we observed a significant drug interaction between ARs and CaC only in patients who underwent hemodialysis with a 72-h interval (Fig. 3b). While a similar trend was observed in patients with a 48-h interdialysis interval, the difference did not reach a significant level (*p* = 0.118, Fig. 3a). This finding may be explained by the smaller sample size for the 48-h interval (*n* = 36) compared to the 72-h interval (*n* = 72). Indeed, the differences in mean increase of serum inorganic phosphorus level (pre-HD vs. post-HD) were comparable for the 48- and 72-h interdialysis intervals (1.9 vs. 1.3 mg/dL and 2.3 vs. 1.6 mg/dL, respectively). In addition, our multivariate analysis detected interdialysis interval of 72 h as a statistically significant covariate for the magnitude of increase in serum inorganic phosphorus concentration during the interdialysis interval after adjusting concomitant use of AR, sex, age, dry weight, daily dose of CaC, and residual GFR (Table 2). These data appear to support an idea that interdialysis interval would be an important factor for the interaction between AR and CaC. Nevertheless, because the present study is a retrospective in design, it is difficult to rule out the multicollineality by only evaluating heterogeneity and internal correlation in the selective variables. Therefore, we cannot draw a conclusive statement regarding the positive correlation between interdialysis interval and hypophosphatemic effects in patients receiving AR and CaC.

There is a large inter-patient variability in serum inorganic phosphorus concentration irrespective of hemodialysis interval and co-administration of ARs, because serum inorganic phosphorus concentration is controlled not only by the extent of intestinal absorption of dietary phosphate but also by parathyroid hormone level, acidosis that causes phosphorus shift from the intracellular to serum compartment, and other factors. In this context, it may be important to employ a study design that allows pair-wise comparisons of data in the same patients. If we had compared the pre-HD and post-PD serum inorganic phosphorus concentrations obtained from different patients, we might have failed to detect a significant difference.

The attenuation of hypophosphatemic effects of CaC by ARs is most likely due to reduced phosphate binding capacity of CaC by ARs in the upper gastrointestinal tract. Precipitated CaC formulations are known to show pH-dependent dissolution rates [12]. Co-administration of ARs with CaC would slow the dissolution of CaC formulation, consequently releasing smaller amount of calcium ions to bind phosphate, compared to when CaC is administered alone. In this context, we examined whether there is any difference among ARs in terms of the extent of interaction with CaC, because Hirschowitz et al. [18] reported that PPIs inhibited gastric acid secretion to a greater extent than H_2_RAs. In our study, 63 patients received a PPI and 10 received an H_2_RA. Multiple comparisons suggested that the magnitude of increase in serum inorganic phosphorus level during the interdialysis duration was comparable between different PPIs (Fig. 4). We were not able to draw any conclusion regarding the difference in AR-CaC interaction between PPIs and H_2_RAs, because the number of patients receiving H_2_RAs was much smaller than those receiving PPIs. As a logical extrapolation based upon the results of the present study, a concomitant use of antacids containing either aluminum or magnesium hydroxide and the combination thereof (e.g., Maalox^®^) with CaC may also affect the hypophosphatemic effects of CaC. However, no patients receiving antacids with CaC were retrieved from our electronic medical records. The most plausible reason for this finding would be that aluminum containing antacids are contraindicated for hemodialysis patients.

If co-administration of ARs with CaC attenuates the hypophosphatemic effects of CaC, it may also affect serum calcium homeostasis. Hyperphosphatemia is known to trigger hyperparathyroidism via the physicochemical inverse equilibrium between serum phosphorus and calcium concentrations [19] and subsequently increase the risk of hip fracture by 4 to 5 times in chronic hemodialysis patients compared to the general population [20]. Although we found a significant difference in the change in albumin-corrected serum calcium concentration between patients receiving CaC alone and those receiving CaC with ARs (Fig. 4), the difference (0.3 mg/dL) was small and clinically irrelevant. In addition, we found no significant difference in incidence of fracture events after initiating chronic hemodialysis between patients treated with CaC alone and those with CaC and AR [5/23 (22%) and 19/85 (22%), respectively]. Further studies with larger numbers of patients are required to conclude the effects of AR on bone complications in hemodialysis patients. Indeed, there are reports indicating that PPIs per se potentially increase fracture risk in the general population [21, 22].

The present study has several limitations that are inherent to the study design. Firstly, no information was available regarding the patients’ adherence to medications and dietary consumption of phosphorus and calcium during the study period. Secondly, plasma concentrations of parathyroid hormone were measured in only less than 15% of patients. Thirdly, it remains unclear whether our data obtained from hemodialysis patients receiving CaC may be extrapolated to those receiving another type of calcium salt phosphate binder, calcium acetate. While calcium acetate is approved in the United States and Europe, it is not approved as an ethical drug in Japan. Fourthly, because the observation period of our study was short (median [interquartile range]: 38 [17, 68] days), it remains to be determined if our findings can be extrapolated to an extended observation period. Finally, we were not able to investigate whether a concomitant administration of PPI or H_2_RA with other phosphate binders (i.e., sevelamer hydrochloride, lanthanum carbonate, bixalomer, and ferric citrate) would affect their hypophosphatemic effects. This was simply due to a scarcity of data: only 13 relevant patients were identified in the electronic medical records.

## Conclusion

Concomitant use of ARs, especially PPIs, with CaC may attenuate the hypophosphatemic effect of CaC in patients undergoing chronic hemodialysis. When patients are taking ARs for the prevention of upper gastrointestinal damages (such as peptic ulcer) due to long-term use of low-dose aspirin or other antiplatelet drugs, it may be prudent to choose phosphorus binders other than CaC.

## Abbreviations

AR: Acid reducer
CaC: Calcium carbonate
ESRD: End-stage renal disease
GFR: Glomerular filtration rate
H_2_RA: Histamine H_2_ receptor antagonist
HD: Hemodialysis
MDRD: the Modification of Diet in Renal Disease
PPI: Proton pump inhibitor
PRC: Partial regression coefficient
SD: Standard deviation
SE: Standard error
SPRC: Standardized partial regression coefficient
VIF: Variance inflation factors
